# Advancing a new evidence-based professional in health care: job task analysis for health and wellness coaches

**DOI:** 10.1186/s12913-016-1465-8

**Published:** 2016-06-27

**Authors:** Ruth Q. Wolever, Meg Jordan, Karen Lawson, Margaret Moore

**Affiliations:** Osher Center for Integrative Medicine, Department of Physical Medicine & Rehabilitation, Vanderbilt Schools of Medicine & Nursing, 3401 West End Ave., Suite 380, Nashville, 37280 TN USA; Integrative Health Studies, California Institute of Integral Studies, CIIS Main Building, 4th Floor, 1453 Mission St., San Francisco, 94103 CA USA; University of Minnesota, Center for Spirituality and Healing, MMC 505 Mayo, 420 Delaware Street SE, Minneapolis, 55455 MN USA; Institute of Coaching, McLean Hospital, Faculty member of Harvard University Extension School, 19 Weston Road, Wellesley, 02482 MA USA

## Abstract

**Background:**

The pressing need to manage burgeoning chronic disease has led to the emergence of job roles such as health and wellness coaches (HWCs). As use of this title has increased dramatically, so has the need to ensure consistency, quality and safety for health and wellness coaching (HWC) provided in both practice and research. Clear and uniform role definitions and competencies are required to ensure appropriate scope of practice, to allow best practices to emerge, and to support the implementation of well-designed, large scale studies to accumulate a rigorous evidence base. Since the nascent field is replete with heterogeneity in terms of role delineations and competencies, a collaborative volunteer non-profit organization, the National Consortium for Credentialing Health and Wellness Coaches (NCCHWC), has been built over the past six years to support professionalization of the field.

**Methods:**

In 2014, a professionally led Job Task Analysis (JTA) was conducted with 15 carefully selected subject matter experts (SMEs) with diverse education and professional backgrounds who were practicing HWC in a wide variety of settings. After establishing a thorough list of specific tasks employed during HWC, the expert panel discussed the knowledge and skills necessary to competently perform the tasks. Subsequently, a large validation survey assessed the relative importance and frequency of each identified job task in conducting HWC.

**Results:**

The JTA identified 21 job tasks as essential to HWC. In the subsequent validation survey, 4026 practicing health and wellness coaches were invited to rate each of the 21 job tasks in terms of their importance and frequency. A response rate of 25.6 % provided a diverse sample (*n* = 1031) in terms of background, and represented a wide variety of training programs from academia, industry, the private sector and associations. Per best practices, the subset of practicing HWCs (*n* = 885) provided importance and frequency ratings to be used to calculate task and domain weights that can serve as a foundation for a NCCHWC national certification examination.

**Conclusions:**

This JTA provides a significant step forward in the building of a clear and consistent definition of HWC that will allow for uniform practice standards and enable more stringent methodology to evaluate this promising approach within evidence-based medicine.

## Background

The need to prevent, ameliorate, or treat lifestyle-related disease has led to the emergence of new roles in health care such as health and wellness coaching (HWC). HWC increases patient activation and health-related self-efficacy [1–4]. Furthermore, three published reviews of HWC suggest its effectiveness in multiple psychosocial variables, behavioral outcomes, and biological markers of chronic disease [5–7]. However, all three reviews note the significant limitation of the literature resulting from variability in the use of the terms health and/or wellness coach. In addition to impeding further study of the field, this variability leaves health care stakeholders and the public confused [8]. To integrate HWC into mainstream health care, a rigorous evidence base founded upon a clear and consistent job definition, scope of practice, and demonstrated competencies, is vital.

Over the past six years, a collaborative volunteer non-profit organization, the National Consortium for Credentialing Health and Wellness Coaches (NCCHWC), was formed to professionalize HWC. Over the past two years, NCCHWC has used best-practice processes to conduct and validate a Job Task Analysis (JTA). Drawing the test specifications from the validation study, the NCCHWC is building a national certification exam. A well-recognized credential founded on best-practices will provide a clear and consistent definition of HWC, with uniform practice standards, to enable more stringent methodology to evaluate this promising approach within evidence-based medicine.

### A heterogeneous literature base for health & wellness coaching

While there are over 350 articles in the peer-reviewed literature describing health or wellness coaching interventions, careful scrutiny reveals enormous heterogeneity in use of the terms “health” or “wellness” coach. Job definitions and the interventions under evaluation run the gamut from minimally trained undergraduate students phoning patients to review and problem-solve homework assigned in a structured chronic disease management program [9], to volunteers with 16 h of training who review with patients the importance of managing chronic conditions [10], to highly-trained health professionals with up to 100 h of additional coach-specific training who use specific processes founded on evidence-based behavior change theories [4, 11, 12]. “Health” or “wellness” coaching is also used to refer to technology interventions without interpersonal communication [13], and to individuals providing interventions that completely lack description [14].

In light of this variability, multiple calls for a consensus-based definition of HWC were made in order for comprehensive and systematic reviews to help establish the evidence base for the effectiveness of HWC and set the stage to identify best practices [2, 7, 8]. The field’s need for a consensus-based definition led to a systematic review of the medical literature on HWC, with the explicit objective of empirically clarifying the operational definition of HWC as it was emerging in the peer-reviewed literature:

*A patient-centered approach wherein patients at least partially determine their goals, use self-discovery or active learning processes together with content education to work toward their goals, and self-monitor behaviors to increase accountability, all within the context of an interpersonal relationship with a coach. The coach is a health care professional trained in behavior change theory, motivational strategies, and communication techniques, which are used to assist patients to develop intrinsic motivation and obtain skills to create sustainable change for improved health and well-being* [15].

The systematic review noted that of the 284 articles reviewed at the time, less than a third described the coaching methods used during the interventions studied, hindering a full evaluation of the effectiveness or contribution of specific methods, and replication in other studies. Hence, to build a robust evidence base on HWC, the tasks conducted by a health and wellness (HW) coach must be well-defined and consistently applied in order to support stringent methodology.

### Establishment of emerging professions

An emerging profession starts with pioneers who develop and test a new competency set and establish applications across different contexts. This phase is followed by innovators who refine these competencies and collect evidence over time, before reaching a maturity phase suited to standardization, credentialing, and eventually licensure. Best practices for delineating emerging job roles include the conduct of a practice analysis, or JTA, followed by a validation survey of the JTA completed by individuals practicing that particular profession or role [16, 17]. The JTA clarifies the real-world content of a job, as well as the requirements necessary for those who perform the job. Hence, the JTA is used to create accurate and valid job descriptions, while defining the tasks, knowledge, and skills that an individual must have to conduct that job at a minimally competent level [18, 19].

Once established, necessary training curricula can be articulated, to delineate valid entry-level skills and basic job requirements. The JTA is based on input from selected practicing coaches, who are in the best position to describe the requirements and content of their work, as compared to educators or theorists more removed from the field [16–19]. The JTA process also presumes that use of well-constructed scientific inquiry and appropriate statistical methodology will lead to a reliable and valid description of job requirements. Use of validation survey procedures and appropriate psychometric assessments ensure reliability as well as content validity to the JTA [17]. Ultimately, the validation survey lays the foundation of the blueprint for a certification exam that tests the applied knowledge required to carry out the tasks as specified in the JTA. Professional certification is thus obtained when a person passes an examination that adequately and proportionally covers the job as delineated and validated in the job task analysis [16, 17].

## Methods

### Phase I: building the task list

The JTA implemented by the NCCHWC for HWC was facilitated by Gerald A. Rosen, EdD, a consulting psychometrician with 32 years of experience in the design, administration and analysis of credentialing programs. A first step in the JTA was to assemble a small panel of Subject Matter Experts (SMEs) who formed a representative sample of practicing professional HW coaches. Experienced HW coaches were nominated by NCCHWC Board members and invited leaders in the field to serve on the SME panel. Nominees completed a survey indicating information about their background and current practice. The initial pool of 38 invitees was narrowed to 34 to ensure an “in the trenches” experience level. Participants were required to coach at least 10 h per week on average and have a minimum of 100 h of practice post training; since four of the nominees did not, they were removed from the pool.

The demographics, professional background, coach training, and work characteristics of the 34 potential invitees were then considered to create a diverse group. Prior to panel selection, the Board identified the factors that were most important to consider in a creating a diverse panel, including the following: gender (to ensure male representation), ethnicity, age, professional background, training programs attended, source of nomination, amount of coaching experience, current work setting, and the area of the country in which the coach practiced. Stratification was done manually by evaluating different configurations for the panel considering the above criteria. For example, a panel which had no dietician representation was rejected, and a panel which had no coaches under 35 years of age was rejected. The NCCHWC Board reviewed the possibilities and decided by consensus on the most diverse. Stratification resulted in 15 representatives.

In March of 2014, SMEs gathered in Indianapolis for a 2.5-day meeting, where the consultant facilitated a process to define the specific tasks that they perform in coaching sessions. The consultant led the participants in an initial brainstorm wherein they noted in a linear fashion each thing they do during a coaching session. The consultant then led participants back through the tasks again and again, repeatedly asking clarifying questions such as “What happens next?” or “Is there anything else you do during this task?” or “Are there times when you don’t do this?” The process was repeated many times to guarantee completeness of the task list, with the consultant pushing the group to think clearly and concretely until the group met consensus on each included task. Consensus was required that the task is essential to perform HWC, independent of how frequently the task is conducted. The process was collaborative; rather than simply listing and voting on task inclusion, tasks were included in the final list only after consensus was achieved. Panel members emphasized that the sequence of coaching tasks is emergent rather than linear; for most of the process, the needs of the patient dictate the sequence of tasks more than an order that is defined a priori.

After panel members felt confident that all tasks were listed, they discussed the relative frequency and importance of each task. While some specific tasks may be performed only rarely, they are essential to the process of HWC. Other tasks may be done frequently, but are less critical [17]. Panel members then came to consensus on the grouping of tasks into domains. Considering both the frequency and importance of each domain, the SME panel then assigned a domain weight to each task grouping to indicate the domain’s relative contribution to the process. The SME-assigned domain weight served as a comparison point for the domain weights that were later derived from the validation study conducted in Phase II.

The JTA SME panel briefly discussed a list of knowledge and skills necessary to support the tasks. Since time limitations precluded thorough analysis of these, the Board convened a second SME group composed of HWC educators with representatives from 20 different programs in academia, government, associations and the private sector. That SME group was professionally facilitated by a specialist in curriculum development and the process used to clarify and refine the knowledge and skills lists is described elsewhere [20]. Those findings will form the basis for national standards for training and education as well as practical assessment for HW coaches.

The NCCHWC board of directors had the responsibility to review the JTA and approve, raise concerns or request amendments. The board approval process to request amendments required consensus of the board, and full approval by the SME panel. Individual SMEs were sent a copy of the JTA through email by the JTA coordinator, with requested edits in tracked changes. Each SME was asked to provide feedback and approve or reject the edits. Panel members responded to the JTA coordinator, who passed the information on to the board. In the case of rejection by any SME, amendments would be denied or reworked until consensus was reached.

### Phase II: validating the task list

#### The validation survey

The final task list was formatted into a survey through www.surveymonkey.com to be disseminated to a large group of practitioners who could either retain or reject each task. The survey included 13 questions regarding participants’ background and coaching practices. Ten of these questions were brief (age, gender, highest degree, etc.) and included a qualifying question to take the survey; it read, “Are you currently practicing as a HW coach?” Participants that said “no” were not provided further access to the survey. Three of the 13 questions on participants’ background and coaching practices required the participant to select from a lengthy list of responses or write in their own answers. The latter three were thus placed at the end of the survey, after the vital survey questions in case of survey fatigue. There were also open fields for participants to provide general feedback on the process to the NCCHWC.

The vital survey questions related to the importance and relative frequency of each task. Within each domain, the tasks were listed and followed by the question, “How IMPORTANT is each task in your current practice as a HW coach?” Corresponding response options, with assigned Likert ratings, were:

- 1 = Not Important - Performance of this task is not essential for your current job.;
- 2 = Somewhat Important – Performance of this task is minimally essential for your current job;
- 3 = Important - Performance of this task is moderately essential for your current job; and
- 4 = Very Important - Performance of this task is clearly essential for your current job.

The tasks for that same domain were then repeated, followed by the question, “How FREQUENTLY is each task performed in your current position as a HW coach?” Response options for frequency, with corresponding Likert ratings, were:

- 1 = Never;
- 2 = Infrequently - less than monthly;
- 3 = Occasionally - 1–3 times per month; and
- 4 = Frequently – greater than 3 times per month.

For Domain IV, the tasks were listed and followed by a single question: “Do you agree that it is important to include questions related to legal and professional considerations on the certification examination?”

#### The validation sample

The validation sample was obtained through snowball sampling. The survey link to the validation survey was first disseminated through email from six HWC training programs directly to their graduates, as well as to practicing coaches from organizations with internal training programs whose program administrators were asked to extend the invitation to their employees. In addition, board members sent the survey invitation to other contacts with access to multiple coaches (e.g., disease management firms, HW associations such as the National Wellness Institute). All individual recipients of the email invitation were also asked to provide contact information to the NCCHWC for anyone else they knew who was a practicing HW coach. Invitations were then sent to those individuals as well. Four of the six training programs that originally disseminated the invitation sent a reminder regarding the invitation. Graduates from the other training programs, organizations with internal training programs, disease management firms, HW associations and additional individual add-ons received the invitation only once.

#### Data analysis

**Fig. 1:**
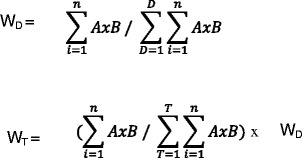
Formulas for Domain Weights and Task Weights. W_D_ = Domain Weight; W_T_ = Task Weight; A = Importance Rating; B = Frequency Rating; D = Domain; T = Task; and n = Number of Responses

Responses to the survey were compiled through Survey Monkey and exported to EXCEL. Descriptive statistics, frequency counts, task weights and domain weights were calculated using EXCEL. Tasks weights and domain weight were based on the importance and frequency ratings, per the formulas presented in Fig. 1 [21].

## Results

### The JTA panel

Sociodemographics, background information and coaching practice characteristics for the 15 SMEs on the JTA panel are shown in Table 1. Significant diversity was achieved in age, educational and professional background, and in practice settings. There was also representation from 17 completed coach training programs, with most SMEs having attended multiple courses. In addition to professionally coaching, over half of the SMEs still maintained clinical licensure in their other professions. Racial and ethnic diversity were quite limited (no Hispanics or African Americans, only one self-identified as Asian/Pacific Native), as was gender heterogeneity (2 of 15 members were male).

**Table 1:** Frequency counts for background and coaching practices of the job task analysis subject matter experts (*n* = 15)

Age ranges (yrs)	26-35	36-45		46-55	56-65		>65
	4	3		4	2		2
Weekly coaching (hours)	<10	10-19		20-29	30-39		40-49
	1^a^	7		5	0		2
Professional Background	Nurse	Psychologist or Clinical Social Worker	Dietician	Exercise Physiologist	Health Educator	Minister	Wellness Coach since graduation
	4^b^	3	1	2	2	1	2
Practice Setting^c^	Medical Outpatient	Insurance Company	Benefits Company	Corporate	Private Practice	Academic Institution	Other^d^
	3	2	3	7	7^e^	4	4

^a^The JTA Coordinator who hosted the event also participated, hence her information was included; her weekly practice was less than 10 h per week as required of the invited SMEs

^b^Two of the nurses had psychiatric specialties

^c^SMEs could select multiple answers

^d^Two practiced in the community (funded through grants), one practiced for the government and one for a fitness, recreation or wellness facility

^e^While seven SMEs coached in private practice settings, only one used private practice as the sole setting for coaching; the other six supplemented their income through private practice

Ten of the 15 SMEs regularly had direct communication with patients’ other care team members. Nine shared their records with other providers. Six used a web coaching platform; eight used an electronic medical record (EMR); and eight were actively involved in coaching with measured outcomes for research purposes.

### Task list

The SME panel identified 16 unique job tasks and provided consensual agreement to the addition of 4 legal and professional considerations tasks (Domain IV) recommended by the JTA consultant. The NCCHWC Board then reviewed the job task list for accuracy and completeness, and identified an additional task that captured the more generative, and creative aspects of coaching that had proved difficult to make concrete during the JTA process: Task - 9: “inviting a client to explore patterns, perspectives, and beliefs that might be limiting health behavior change.” The JTA SME panel unanimously approved the amendment. Lengthy discussions of this task led to careful choice of wording to clarify that coaches *invite* exploration of perspectives and beliefs rather than analyze their roots or origins, as such analysis falls outside the training and scope of HW coaches [22]. See Table 2 for the final task list and domains, along with their validations weightings (described below).

**Table 2:** Job tasks, frequency and importance validation ratings and associated weights (*n* = 885)

Domain I: The tasks that comprise and define this Domain are concerned with the activities that take place in the initial stages of the coaching process. (Domain weight = 25.6 %)	IMP	FREQ	Weight
T-1 Explain the coaching process.	3.32	3.48	5.04
T-2 Obtain information about why coaching is sought, desired outcomes, priorities, personal strengths and challenges.	3.50	3.58	5.44
T-3 Determine if the individual is a candidate for health and wellness coaching.	3.17	3.33	4.59
T-4 Explore motivation and assess readiness for change.	3.67	3.79	6.05
T-5 Jointly create a coaching agreement that includes roles, expectations, practice-specific processes, fees, and frequency, mode and length of sessions.	3.18	3.23	4.48
Domain II: The tasks that comprise and define this Domain are used throughout the Health and Wellness Coaching relationship and are the most central to the coaching process. (Domain weight = 40.7 %)	IMP	FREQ	Weight
T-6 Assist the client in creating a description of their ideal vision of the future.	3.57	3.51	5.45
T-7 Establish or identify the present situation, past history, previous successes and challenges, resources, etc. associated with the client’s vision.	3.56	3.63	5.62
T-8 Explore and evaluate the client’s readiness to progress toward the vision.	3.59	3.63	5.68
T-9 Invite the client to identify and explore patterns, perspectives, and beliefs that may be limiting lasting change.	3.52	3.65	5.58
T-10 Work with the client to establish goals that will lead to the vision.	3.75	3.80	6.21
T-11 Work with the client to develop a series of steps that will lead to the achievement of client-selected goals.	3.74	3.83	6.23
T-12 Elicit the client’s commitment to and accountability for specific steps.	3.63	3.74	5.92
Domain III: The tasks and knowledge that comprise and define this Domain are concerned with the activities that address the client’s evaluation and integration of progress. (Domain weight = 30.7 %)	IMP	FREQ	Weight
T-13 Collaborate as the client evaluates success in taking steps and achieving goals.	3.60	3.73	5.85
T-14 Work with the client to maintain progress and changes.	3.66	3.77	6.00
T-15 Collaborate as the client re-assesses goals and makes modifications based on personal decisions and progress made.	3.67	3.70	5.90
T-16 Assist the client in articulating learning and insights gained in the change process.	3.58	3.67	5.72
T-17 Work with the client to develop a post-coaching plan to sustain changes that promote health and wellness.	3.55	3.37	5.21
Domain IV: The tasks that comprise and define this Domain underlie all Health and Wellness Coaching practice and the professional behavior of coaches. (Domain weight = 5.0 %)			
T-18 Health and Wellness Coaches practice in accordance with applicable laws and regulations.			
T-19 Health and Wellness Coaches practice in accordance with accepted professional standards and within the limits of their scope of practice.			
T-20 Health and Wellness Coaches practice in accordance with the accepted standards of professional ethics.			
T-21 Health and Wellness coaches engage in a continuous process of training and education to become more proficient in their practice and to ensure that their practice-related knowledge and skills remain current.			

Scores based on 1–4 Likert scales. For Importance (IMP): 1 = Not Important (task is not essential); 2 = Somewhat Important (task is minimally essential); 3 = Important (task is moderately essential); 4 = Very Important (task is clearly essential for the current job). For Frequency (FREQ): 1 = Never; 2 = Infrequently (less than monthly); 3 = Occasionally (1–3 times per month); and 4 = Frequently (weekly)

The SME panel then grouped the tasks into four domains, as follows:

1. Tasks in the initial stages of the coaching process;
2. Tasks most central to the coaching process;
3. Tasks that address the client’s evaluation and integration of progress; and
4. Legal and professional considerations.

### The validation sample

Electronic invitations were extended to 4026 emails requesting participation in the survey; 1031 individuals responded delivering a response rate of 25.6 %. Ninety-two percent were female; other characteristics are presented in Table 3. Seventy-eight percent of the sent invitations were followed by a reminder; 85 % of those who responded to the survey invitation (*n* = 1031) had received a reminder. Of those who responded, 885 reported that they were actively coaching, and thus their responses were utilized to validate the task list, consistent with JTA best practices [17]. When asked about coach training programs completed, 746 (285 of the total sample of 1031 omitted the question as it was marked “optional”) indicated they obtained their coach training by completion of the following: 15 university-based training programs; 18 private sector HWC programs; 23 private sector life coaching programs; five private sector executive coaching programs; and seven training programs offered through fitness or wellness associations (e.g., American College of Sports Medicine, National Wellness Institute, YMCA). In addition, there were eight degrees or certifications that participants believed qualified as HW coach training. They ranged from advanced practice degrees in holistic nursing and various types of psychology to holistic health programs or fitness training.

**Table 3:** Percentages (*n*) for background and coaching practices of validation survey participants (*n* = 1031)

Age (years)^a^	≤25	26-35		36-45		46-55		56-60		>61
	1.5 % (14)	14.3 % (131)		20.8 % (191)		32.7 % (300)		17.9 % (164)		12.8 % (117)
Educational Background^b^	One year certificate no degree		Associate’s degree		Bachelor’s degree		Master’s degree		Doctoral degree	
	2.5 % (23)		3.3 % (30)		40.3 % (368)		45.6 % (417)		8.3 % (76)	
Years practicing HWC^b^	<1		1-4		5-10		11-15		>15	
	17.8 % (163)		50.2 % (459)		26.4 % (241)		3.2 % (29)		2.4 % (22)	
Geographic area^a,c^	West		Midwest		South		Northeast		Multiple States &/or Other Nations	
	20.0 % (183)		29.0 % (266)		28.8 % (264)		15.7 % (144)		6.5 % (60)	
Primary Setting for Coaching Practice^d,e^	Medical/Clinical Facility	Insurance Company	Coaching Services Contractor	Employee Health, Fitness & Wellness	Independent Contractor/Self-Employed	Health Club/Fitness Facility	Government or Military	University/Academic	Community-based Facility (churches, rec centers, etc.)	Other
	24.6 % (186)	5.3 % (40)	8.2 % (62)	12.8 % (97)	37.9 % (286)	3.7 % (28)	2.1 % (16)	2.4 % (18)	2.1 % (16)	0.08 % (6)

^a^114 participants did not answer (*n* = 917)

^b^117 participants did not answer (*n* = 914)

^c^First author used government website [29] to categorize participant’s practice location into geographic areas

^d^276 participants did not answer (*n* = 755)

^e^Participants could select multiple categories

The mean Importance and Frequency ratings for each job task can be found in Table 2, with the listing of the tasks. Table 2 also contains the task weights and domain weights based on the survey responses. For the first three domains, the domain weights originally proposed by the JTA panel were remarkably similar to those of the validation survey. Comparatively, the domain weights for the JTA panel versus the validation survey participants, respectively, were 25.0 % versus 25.6 % for Domain I, 40.0 % versus 40.7 % for Domain II, and 30.0 % versus 28.7 % for Domain III. The JTA panel proposed a weight of 5 % for Domain IV. In the validation survey, the single question relevant to Domain IV (Legal and Professional Considerations) was answered in the affirmative by 95 % of the sample.

## Discussion

This paper describes the process and findings from the NCCHWC JTA. A panel of SMEs diverse in age, professional background, health coach training, practice setting and geographic spread produced a list of tasks that delineate what a HW coach does. The 21 tasks presented in the final JTA were strongly validated by a large sample of practicing HW coaches. Except for race and gender, the validation sample was also highly diverse in terms of sociodemographics, professional and training background and current practice settings. In fact, the wide diversity in professional background and training further supports the need to have a clearly defined minimal standard of competencies.

The response rate recommended for sound JTA validation studies is between 10 and 20 % [23]. While we can not know with certainty, our response rate of 25.6 % from email (about half received a reminder) may indicate enthusiasm in the field for a national standard. Alternatively, it may be a reflection of close ties between HW coaches and their training programs.

### Interpretation of the JTA

The JTA enables the NCCHWC to build an accurate, valid and legally defensible certification examination [17, 18]. The domain and task weights generated through the JTA process set the foundation for the specifications for the first national certification exam for HW coaches. The typical criteria for the acceptance of Tasks range from a mean Importance rating of 2.0 (lenient) to 3.0 (stringent) [23]. As seen in Table 2, all mean Importance ratings exceeded 3.0 and, therefore, all of the tasks merit inclusion in the final certification examination specifications.

The decision to retain or reject tasks in Domain IV, which address legal, ethical, and professional considerations, was made in a different manner. Survey respondents were asked if they thought it important for the certification examination to include questions pertaining to such matters. Since approximately 95 % answered in the affirmative, Domain IV tasks also merit inclusion in a knowledge certification examination. Five percent was retained as the domain weight in the test specifications based on the overwhelming survey respondent support for the retention of Domain IV.

### Tasks of a health and wellness coach

With the newly validated JTA, there is now a clear role delineation to be followed by those practicing as HW coaches. The tasks in the JTA make it clear that HW coaches do not diagnose, interpret behavior or beliefs, or clinically advise patients on what to do. Instead, HW coaches follow specific procedures that elicit personally meaningful HW goals from the patient, and use a number of communication and experiential learning techniques to aid the patient in self-discovery and generation of solutions. Importantly, HW coaches help the patient strengthen motivation, establish systems of accountability and integrate their own learning in how to best shift their behavior in the context of their own life circumstances.

While there certainly are many gaps in the medical system that need to be addressed through new roles, use of the term “coach” for other roles is confusing to patients and providers. According to this validated JTA, coaches are not “navigators” who tell a patient how to work through the complexities of the health care system. They are not a type of physician extender who calls patients to remind them to take their medications or come to appointments. Nor are they administrative personnel who also have good interpersonal skills. All of these roles may be exceptionally important, but a different term is needed to avoid confusion in the literature, and indeed among the public.

### Developing a certification examination

NCCHWC used best-practice processes to delineate the role of HW coaches. The professionally-led JTA produced 21 tasks that were validated by a national survey of 1031 respondents, of whom 885 were actively practicing HW coaches whose task ratings were included in the task and domain weights. Survey results revealed a high degree of support for all tasks, and thus all merit inclusion in the test specifications for the NCCHWC certification exam. The fact that the validation sample in itself represented such diversity in terms of educational background and coaching training underscores the need to set at least a minimal bar for background education and for professional competencies specific to HW coaching.

Consistent with the majority of health professional organizations, the NCCHWC will administer only a written exam since the delivery of practical and oral skills examinations has been criticized for low reliability and validity while simultaneously incurring high administration costs [24]. In order to be eligible for the NCCHWC professional certification exam, a minimal skill set must be obtained and documented. Exam applicants must first complete pre-requisites that demonstrate competency in the minimal knowledge and skills necessary to conduct the role of HW coach as defined by the JTA. These pre-requisites include satisfactory completion of healthy lifestyle education, training from an NCCHWC-accredited HW coach training program, practical skills assessment, and documentation of practical experience [20]. As pre-requisites are updated, they will be listed on NCCHWC.org.

Using the tasks identified and validated in the JTA, the preparation of a certification examination has begun, with the content covered according to the relative weights obtained in the validation survey. The written examination will include approximately 150 multiple-choice questions using the blueprint foundation established in the JTA. To begin preparing items for the exam, 19 item writers who are also actively practicing coaches were recruited and underwent a 90 min training by the JTA consultant. The training emphasized that examination test questions which follow best practices must include the right proportion of question types (i.e. assessment of knowledge and recall, application and interpretation, as well as problem solving and evaluation skills).

Following training, item writers were assigned JTA domains in which to write questions or items. Potential questions were created and submitted to the JTA consultant for review. If appropriately structured, the questions were then submitted to a pool of questions to be reviewed by a smaller Item Review Panel comprised of members with no vested interest in any particular coach training program. The Item Review Panel will convene to make a final determination once an exam administration partner has been chosen. The certification examination will require ongoing assessment to ensure content validity and be certain that it remains relevant to HWC practices.

### Limitations

Despite building collaborative relationships over a 6 year period and using best-practice processes to delineate the specific tasks of a HW coach, there are at least four significant limitations to the process. First, the rapidly growing field of HWC has myriad players, some of whom have vested interests in maintaining their own definition rather than contributing to a consensus approach. While the NCCHWC welcomes participation of new and existing groups, and indeed continues to grow, it will never be possible to have all parties in complete agreement. Second, the SME panel that formed the JTA was diverse in terms of myriad coaching-related variables and background characteristics, but quite limited in terms of racial and ethnic diversity. In addition, the validation survey did not ask participants their race or ethnicity. While this is common practice for role delineation studies [25–27], there is no published sociodemographic data on practicing HW coaches. Hence, we have no way to evaluate how representative our sample is of the field itself. Third, non-probability sampling for surveys can result in a number of biases [28]. First, the sampling was limited to those with online access who had completed specific training programs that sent the survey invitations to their graduates. Although we reached out to many programs, there was self-selection in whether or not programs responded. There was also self-selection in which individuals responded. Since 78 % of the respondents had received a reminder email, those that did not respond may have been less interested in a national certification or simply forgot about the original email. In addition, the numbers reported regarding the validation sampling process may not accurately reflect those who received the invitation. We did not ask the training programs to collect data on how many invitations had incorrect emails and were returned. Moreover, it is likely that some invitees received more than one invitation since some invitees had completed more than one training program. Fourth and finally, the field is not yet mature enough for licensure. While certification and licensure both require individuals to demonstrate a certain level of knowledge and ideally ability, only licensure refers to the legal rights of being able to practice a profession in a given locale. However, licensure is practically driven by economic and systemic priorities in any given state; until there is a reasonable volume of consistently credentialed practitioners in the field, licensure is unlikely to be financially viable or practical. Although a national certification will begin to create uniformity that allows for better research and accumulation of evidence to move toward best practices, regulation of use of the term HW coach and enforcement of the scope of practice are left to the field at large to self-police.

## Conclusion

The NCCHWC has garnered the support of numerous stakeholders with an investment in professionalizing the field of HWC, and will continue to welcome others. In fact, since the original submission of this paper, 45 programs have applied to the NCCHWC for transition accreditation approval (L.A. Webster, Administrator of NCCHWC, personal communication, 1/11/16). The NCCHWC has thus far put forth six years of volunteer effort in collaborative self-regulation to build a national standard. The first JTA is complete, with content validity well supported through a large validation survey. The development of the first national certification examination is underway, and guidelines for certification eligibility have been proposed [20]. If well-adopted, this JTA along with a high quality training program accreditation process, will lead to a clear and consistent definition of HWC, with uniform practice guidelines, that raises standards for HWC. The emergence of such standards will allow for more stringent methodology to evaluate both the efficacy and effectiveness of HWC.

## Abbreviations

HW, health and wellness; HWC, health and wellness coaching; HWCs, health and wellness coaches; JTA, job task analysis; NCCHWC, National Consortium for Credentialing Health and Wellness Coaches; SME, subject matter expert
